# Blood serum metabolome of atopic dermatitis: Altered energy cycle and the markers of systemic inflammation

**DOI:** 10.1371/journal.pone.0188580

**Published:** 2017-11-27

**Authors:** Aigar Ottas, Dmytro Fishman, Tiia-Linda Okas, Tõnu Püssa, Peeter Toomik, Aare Märtson, Külli Kingo, Ursel Soomets

**Affiliations:** 1 Department of Biochemistry, Institute of Biomedicine and Translational Medicine, University of Tartu, Tartu, Estonia; 2 Faculty of Science and Technology, Institute of Computer Science, University of Tartu, Tartu, Estonia; 3 Quretec OÜ, Tartu, Estonia; 4 University of Tartu, Tartu, Estonia; 5 Department of Food Hygiene, Estonian University of Life Sciences, Tartu, Estonia; 6 Clinic of Traumatology and Orthopedics, Tartu University Hospital, Tartu, Estonia; 7 Department of Dermatology, University of Tartu; Clinic of Dermatology, Tartu University Hospital, Tartu, Estonia; National Research Council of Italy, ITALY

## Abstract

Atopic dermatitis is a chronic inflammatory disease which usually starts in the early childhood and ends before adulthood. However up to 3% of adults remain affected by the disease. The onset and course of the disease is influenced by various genetic and environmental factors. Although the immune system has a great effect on the outcome of the disease, metabolic markers can also try to explain the background of atopic dermatitis. In this study we analyzed the serum of patients with atopic dermatitis using both targeted and untargeted metabolomics approaches. We found the most significant changes to be related to phosphatidylcholines, acylcarnitines and their ratios and a cleavage peptide of Fibrinogen A-α. These findings that have not been reported before will further help to understand this complex disease.

## Introduction

Atopic dermatitis (AD) is a chronic inflammatory skin disease that often exhibits characteristic symptoms in the early childhood and can affect up to 20% of children in developed countries and 1–3% of adults in developed countries [1, 2]. Various genetic and environmental factors including allergens contribute to the onset and course of the disease but more specifically dendritic cells have been proven to represent a central part in the management of inflammation [3]. The upregulation of certain T helper 2 cytokines like interleukin-4 and interleukine-13 can also have an effect on the skin barrier in addition to mutations in the filaggrin gene [4] that encodes the protein responsible for the prevention of water loss in the skin and protection against microbial pathogens and other irritants [5]. Other notable biomarkers that correlate with the disease are serum thymus and activation-regulated chemokine, E-selectin, serum cutaneous T-cell attracting chemokine, macrophage-derived chemokine, interleukin-18 and lactate dehydrogenase [6]. Initial studies in the field of metabolomics on atopic dermatitis have been published at the beginning of this decade. First, Assfolg and collaborators demonstrated using NMR higher levels of creatine, creatinine, citrate, 2-hydroxybuturate, formate, dimethylglycine and lactate in the urine of AD children. However, the levels of betaine, alanine and glycine were lower in urine samples when compared to controls [7]. The increase in lactate levels is indicative of activated anaerobic glycolysis, while the decrease in alanine hints at the increased rate of gluconeogenesis. Another study by Hotze *et al* describes an altered metabolite profile of lipids in AD patient’s blood serum samples. Most drastic changes could be noted in the elevated levels of glycerophospholipids (PC) in particular phosphatidylcholine acyl-alkyl C38:1, lysophosphatidylcholine acyl C26, lysophosphatidylcholine acyl C28:0 [8]. A third study by Huang *et al*, using ultra high performance liquid chromatography tandem mass-spectrometry, reported the reduction of concentration of glycine and taurine conjugated bile acids while the levels of cholic acid and chenodeoxycholic acid were increased in children’s serum suffering from AD. In addition, the increase of free fatty acids was also noted in the study [9]. Overall it can be pointed out that the work done in the field of metabolomics on atopic dermatitis is still in its beginning phase and a lot of the results need further confirmation and explanation. In this study we aim to expand on the understanding of biochemistry behind atopic dermatitis using both the targeted and untargeted methods used in metabolomics. The findings may lead to a better explanation of the underlying mechanisms in atopic dermatitis.

## Materials and methods

### Volunteer recruitment

The adult patients with atopic dermatitis were recruited between 2013–2015 from the University Hospital of Tartu at the Clinic of Dermatology stationary department when they first arrived and before treatment began whereas the controls were either from the same clinic or from the Clinic of Traumatology and Orthopaedics. No count was kept on the number of volunteers who refused to participate in the study. Since participants only needed to give a sample at the beginning of the study, the dropout rate could not be calculated. Volunteers, who had diabetes or any comorbid skin diseases, were excluded from the study. The patients were all diagnosed with severe atopic dermatitis and had various other allergies and asthma in 3 cases. 13 volunteers with AD and 15 age and sex-matched controls (ages 20–61, 9 men, 19 women) were included in the untargeted analysis measurements and expanded to 25 patients and 24 controls for the targeted analysis (ages 20–55, 12 men, 37 women). Using a wide age range of adult patients ensures that the changes seen are not age-specific and that they can be attributed to atopic dermatitis. The recruited participants were all Caucasians of eastern European descent.

### Blood collection and storage

Blood samples were collected in the morning before breakfast using 5 ml Vacutainer (REF 367614) tubes containing micronized silica particles to accelerate the clotting process. The samples were left at room temperature for one hour, centrifuged at 1300 × g for 20 minutes, the supernatant serum was pipetted into 300 μl aliquots, stored at -80°C until measurement.

### Materials

HPLC grade acetonitrile, water, methanol and formic acid (FA) were purchased from Sigma-Aldrich (Germany).

### Targeted metabolic analysis

An Agilent Zorbax Eclipse XDB C18, 3.0 × 100 mm, 3.5 μm with Pre-Column SecurityGuard, Phenomenex, C18, 4 × 3 mm was used with the Absolute*IDQ* p180 kit (Biocrates Life Sciences AG, Innsbruck, Austria) for the targeted analysis of 188 metabolites, measured on a QTRAP 4500 (ABSciex, USA) in tandem with a 1260 series HPLC (Agilent, USA). The exact protocol for the preparation of the samples is detailed in the Absolute*IDQ* p180 kit’s user manual. In short, the serum samples were thawed on ice, pipetted onto the included 96-well plate (10 μl per sample), internal standards added and derivatized using phenylisothiocyanate. The concentrations of numerous metabolites including acylcarnitines, biogenic amines, amino acids, hexose, sphingolipids and glycerophospholipids were determined using a combination of flow injection analysis and liquid chromatography through a C18 column.

### Untargeted metabolic analysis

The untargeted measurements were made on a 6450 UHD Accurate Mass Q-TOF tandem liquid chromatograph with 100 series quaternary pump (Agilent, USA). The column used was EclipsePlus C18 RHD 1.8μm 2.1 × 50 mm (Agilent, USA). Serum samples were processed according to a protocol by Want *et al* [10]. A quality control sample was prepared by pooling 20 μl of serum from every sample and measured after 5 sample measurements. The samples were measured in a random order using the protocol by Want *et al*[11]. Mass-to-charge (m/z) ratios of interest from the statistical analysis were subjected to fragmentation with identical run parameters to earlier measurements.

### Handling of data and statistics

MetIDQ (BioCrates, Austria) and Analyst (ABSciex, USA) software were used for the calculation of the metabolite concentrations from the targeted analysis. Data from both targeted and untargeted analysis have been analysed and pre-processed using the R programming language version 3.4.2[12] in RStudio 0.98.501. Untargeted analysis measurement data (from positive and negative ionization modes) required processing using the library mzMatch.R [13] where peak picking was applied using XCMS [14], also biological replicate combining, correction of retention time, RSD filtering, QC correcting and filling of gaps. In order to approach normal distribution of data from both experiments, log_10_ transformation was applied, mean subtracted for each data point and divided by the standard deviation. Later, Shapiro-test was used to test for normality. The data were normally distributed in the targeted analysis and not normally distributed in the untargeted analysis. Non-paired t-test was used for targeted analysis and Mann-Whitney Wilcox test was applied to the untargeted analysis data. All p-values were FDR 5% corrected. PCA was used on both targeted and untargeted analysis first to assess that there are no visual batch effects present in the data. Later, PCA plots were generated using differentiating metabolites to confirm if they produce visually distinct clusters of samples that correspond to studied phenotypic groups. PLS-DA plots were generated to confirm the results of PCA plots using statistically significant metabolites.

### Verification of significant metabolites via machine learning

In order to verify the importance of metabolites that were found to be statistically significantly different between phenotypes for the discrimination of patients and controls, three well-known machine learning algorithms were applied: GLMNET [15], PDA [16] and RandomForest [17].

Using metabolites identified by univariate tests as features for supervised models was shown to inflate accuracy of constructed classifiers. This type of bias was called selection bias [18]. Therefore, in order to avoid selection bias but still be able to verify metabolites found to be significant, the three mentioned classifiers were trained on the whole data from both targeted and untargeted analysis using 5-fold cross-validation algorithm [19] repeated 5 times to avoid overfitting. Overfitting is a problem of the model not being able to generalize to unseen data due to the limited size of training set [20].

Average performance was recorded for each classifier separately. Accuracy as a performance metric was used. If classifiers were capable of achieving an average accuracy larger than 0.5 (random choice), it was concluded that it is possible to differentiate between groups of samples. Three classifiers were used to ensure that none of the models has overfitted the data. Then, RandomForest importance measure was used to sort metabolites by their influence on the final model. If metabolites found using statistical tests were indeed important for discriminating between patients and controls, these metabolites must have been used by RandomForest and thus, should have been among the topmost important metabolites.

### Identification of metabolites

The fragmentation spectra from the untargeted analysis were matched to public databases HMDB [21], MassBank [22], METLIN [23], and LipidMaps [24]. Identification was positive when a specific compound’s fragmentation spectra’s peaks and relative heights were comparable to database’s spectrum.

## Results

The untargeted analysis resulted in 6 statistically differing metabolites out of which 3 were identified (S1–S3 Figs)–a peptide DSGEGDFXAEGGGVR and phosphatidylcholine PC(16:0–16:1) /(14:0–18:1) levels in the serum were both significantly higher in AD patients while phosphatidylcholine PC(16:1/20:4) was lower in AD patients’ serum. The targeted analysis yielded a total of 7 metabolites that differ statistically significantly between groups: acetylcarnitine (C2), phosphatidylcholine diacyl C38:5 (PC.aa.C38.5), phosphatidylcholine diacyl C40:5 (PC.aa.C40.5), ratio of short chain acylcarnitines (acylcarnitine–C2, propionylcarnitine–C3) to free carnitine (C0), ratio of acetylcarnitine to free carnitine (C2…C0), fraction of dicarboxylacylcarnitines of the total acylcarnitines (Total.AC.DC…Total.AC) and ratio of esterified acylcarnitines to free carnitine (Total.AC…C0). Statistically significantly different m/z-s from the targeted analysis are given in Table 1 and S4 Fig; metabolites from the untargeted analysis in Table 2 and S5 Fig. No statistically significant changes could be seen in amino acids, biogenic amines, hexoses or sphingolipids in targeted analysis.

**Table 1:** Targeted analysis results from non-paired t-test where atopic dermatitis patients’ serum metabolites were compared to controls.

Metabolite	p-value	AD mean	Control mean
C2	0.026	-0.617	0.397
PC.aa.C38.5	0.026	-0.667	0.142
PC.aa.C40.5	0.026	-0.663	0.195
X.C2.C3. . . .C0	0.026	-0.232	0.737
C2. . .C0	0.026	-0.237	0.736
Total.AC.DC. . .Total.AC	0.026	0.551	-0.353
Total.AC. . .C0	0.036	-0.174	0.698

**Table 2:** Untargeted analysis results from Mann-Whitney Wilcox test where atopic dermatitis patients’ serum metabolites were compared to controls.

Positive mode	m/z	Identification	AD mean	Control mean	p-value
	545.393	unknown	-0.98	0.85	0.0075
	737.735	DSGEGDFXAEGGGVR	0.98	-0.85	0.0075
	780.611	PC(16:1/20:4)	-0.91	0.79	0.0192
	829.891	unknown	-0.91	0.79	0.0192
	641.512	unknown	-0.77	0.67	0.0430
Negative mode	537.507	PC(16:0–16:1) /(14:0–18:1)	0.84	-0.73	0.0351

**Fig 1:**
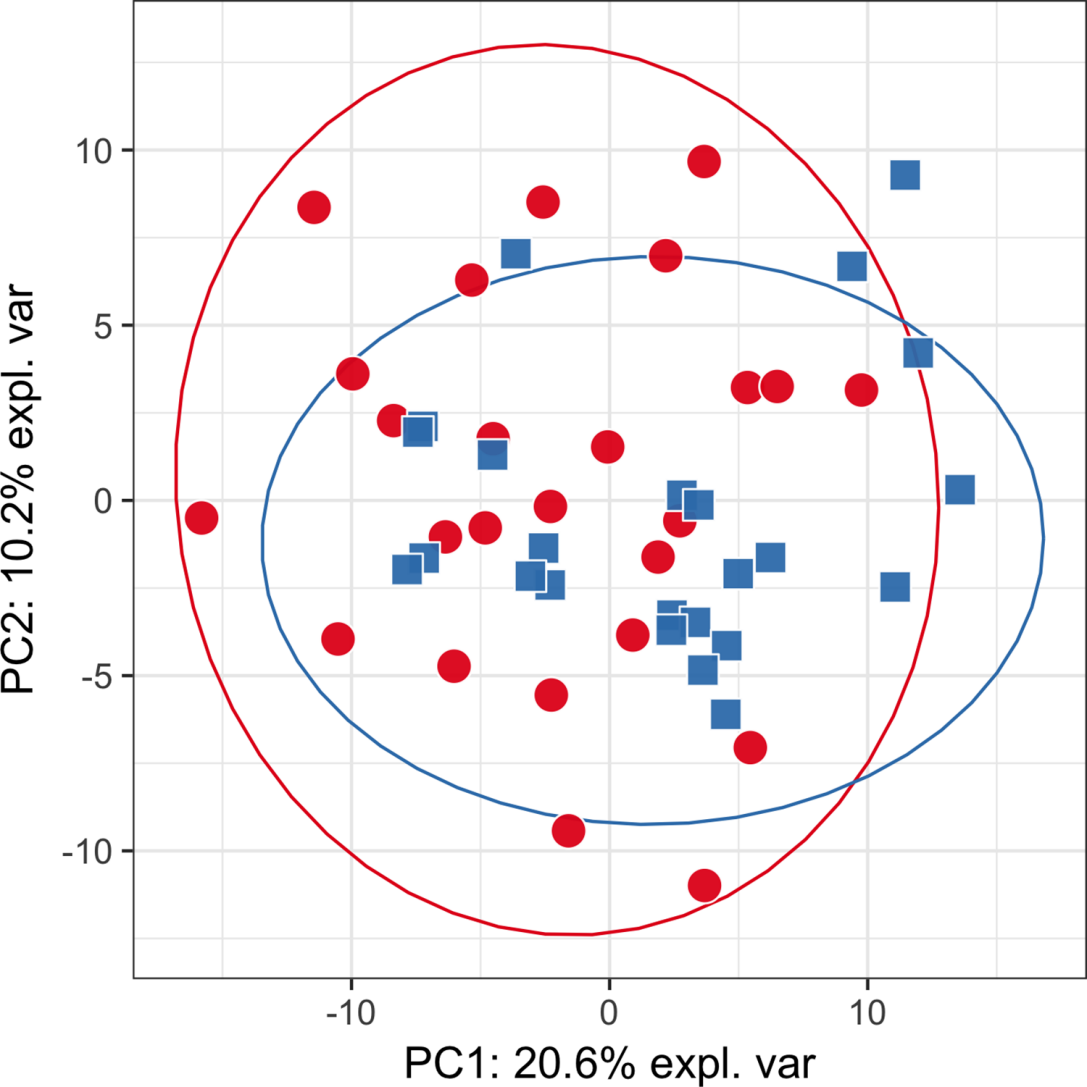
PCA plot for targeted analysis based on the whole data. Red circles—cases; blue squares—controls. Initially, both groups largely overlap, also PCs explain a modest amount of variance.

**Fig 2:**
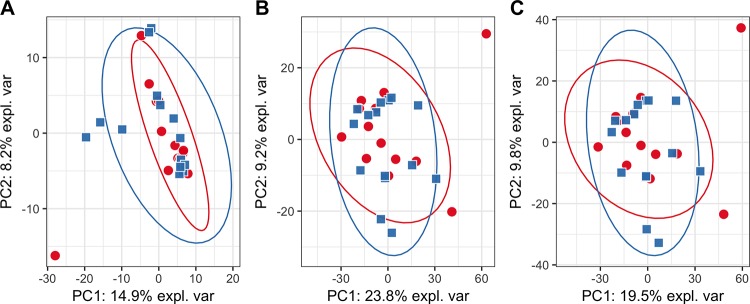
**PCA plot for untargeted analysis based on negative (A), positive (B) and combination of both (C) datasets.** Red circles—cases, blue squares—controls. It can be seen that adding the dataset obtained from negative mode does not explain additional variance in the positive mode dataset, on the contrary—the amount of variance explained by the first and second principle components decreases for the combined dataset, which suggests that there is very little important information in the dataset obtained from negative mode.

**Fig 3:**
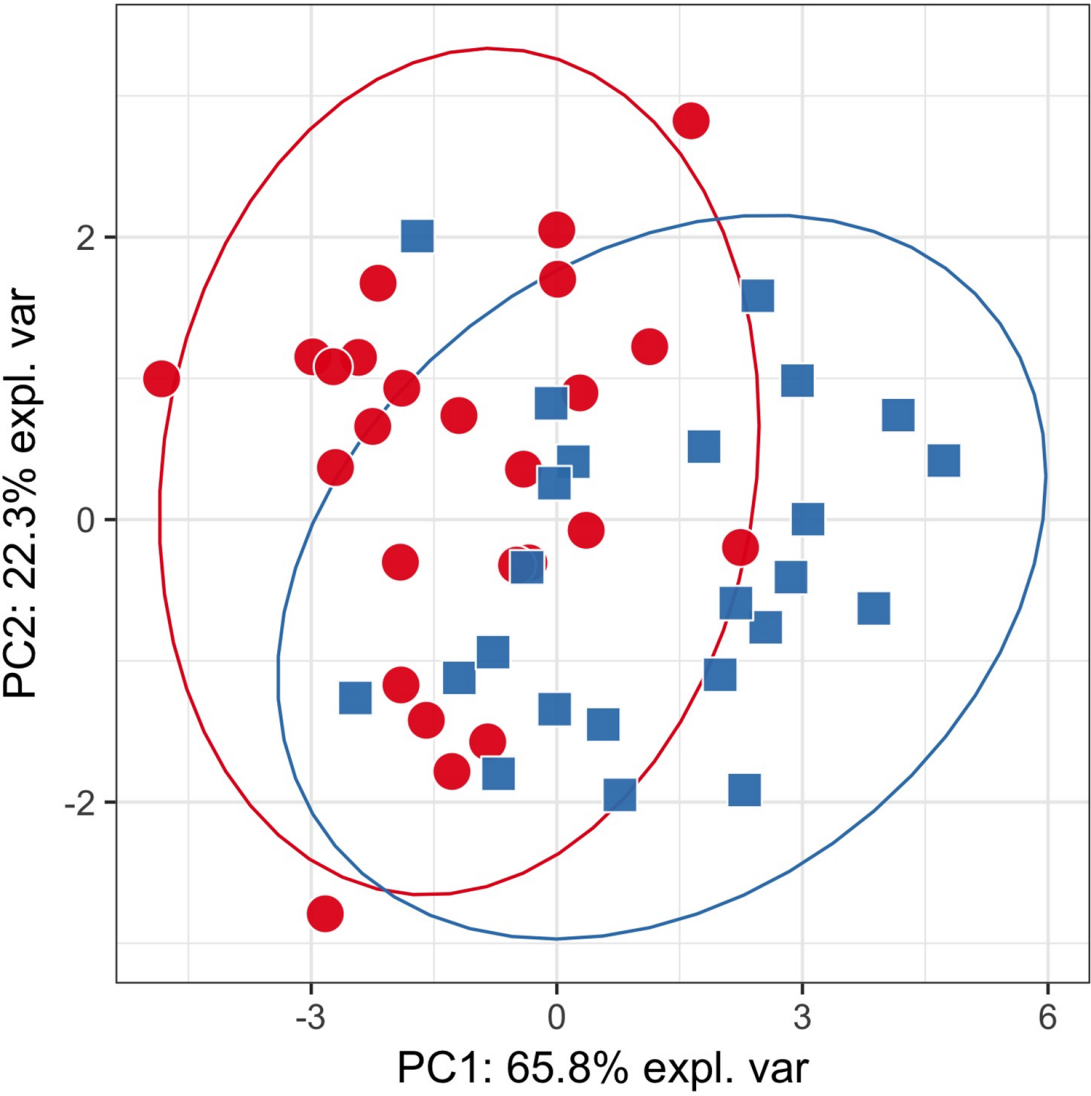
PCA plot for targeted analysis based on the significantly different metabolites. Red circles—cases; blue squares—controls. The groups are visually separable, although there is still a significant overlap between samples. It is important to note that PCs explain much more variance than in Fig 1.

**Fig 4:**
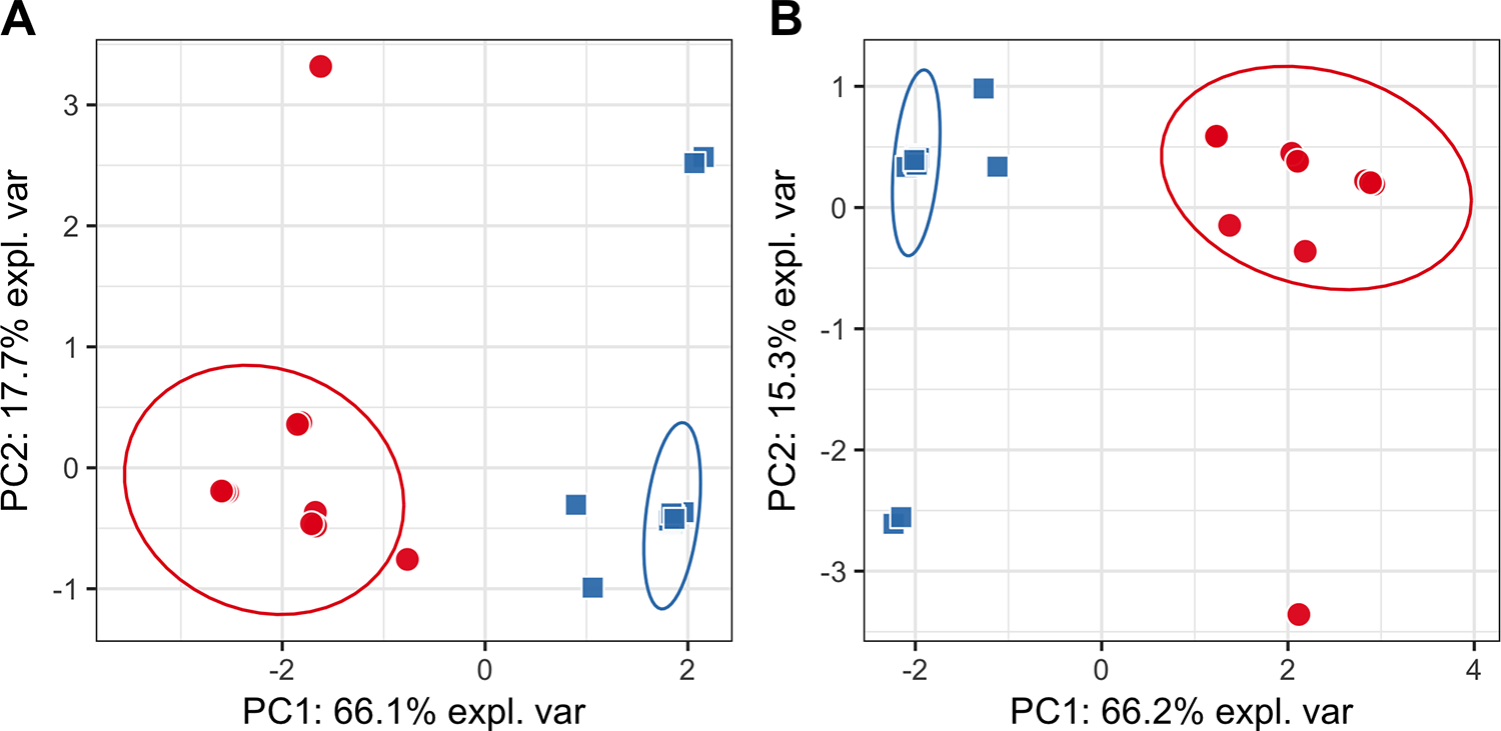
**PCA plots based only on metabolites that were found to be significant in untargeted analysis for positive (A) and combined (B) datasets.** Red circles—cases; blue squares—controls. Points on both plots are visually separable and form very clear clusters that correlate with phenotypes.

PCA plots constructed on the whole data from targeted (Fig 1) and untargeted analysis (Fig 2) did not show any hidden batch effects that could bias metabolite identification. The separate clustering of phenotypic groups could also not be seen, suggesting that the use of simple linear models will not differentiate classes. PCA plots generated using only statistically significantly different metabolites produced visually distinct clusters of samples that correspond to studied phenotypic groups (Figs 3 and 4). PLS-DA plots show a clear clustering of samples, similarly to PCA plots using only statistically significantly different metabolites (S6 and S7 Figs).

**Fig 5:**
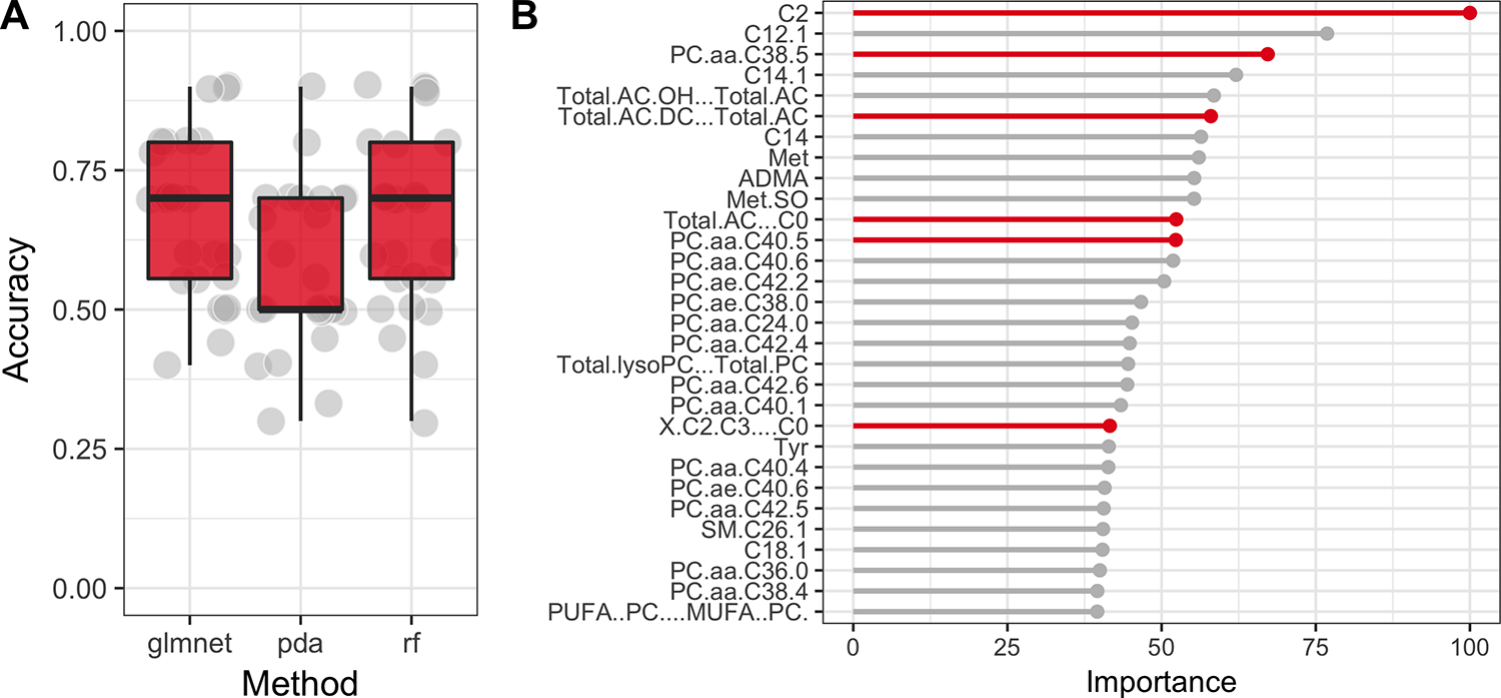
A Average classification performance (accuracy) of three distinct classifiers (rf = Random Forest, pda = Pennalised Discriminant Analysis and glmnet = Lasso and Elastic-Net Regularized Generalized Linear Model) on data from targeted analysis. The performance was measured with cross-validation algorithm over 5 folds and 5 repetitions. Average performance reaches 70%–75%, which suggests that metabolites are indeed capable of distinguishing between cases and controls. B Top 20 most important metabolites used by RandomForest classifier. Metabolites identified as significantly different in targeted analysis are highlighted. We can see that indeed almost all metabolites (except for C2…C0) identified as significantly different are in the top of the list.

In targeted analysis, machine-learning models achieved an average performance close to 75%, suggesting that it is possible to differentiate between cases and controls using metabolites measured in targeted analysis (Fig 5A). Importantly, metabolites identified as significant in targeted analysis appear in the top of the list of metabolites that were most influential for RandomForest classifier (Fig 5B).

**Fig 6:**
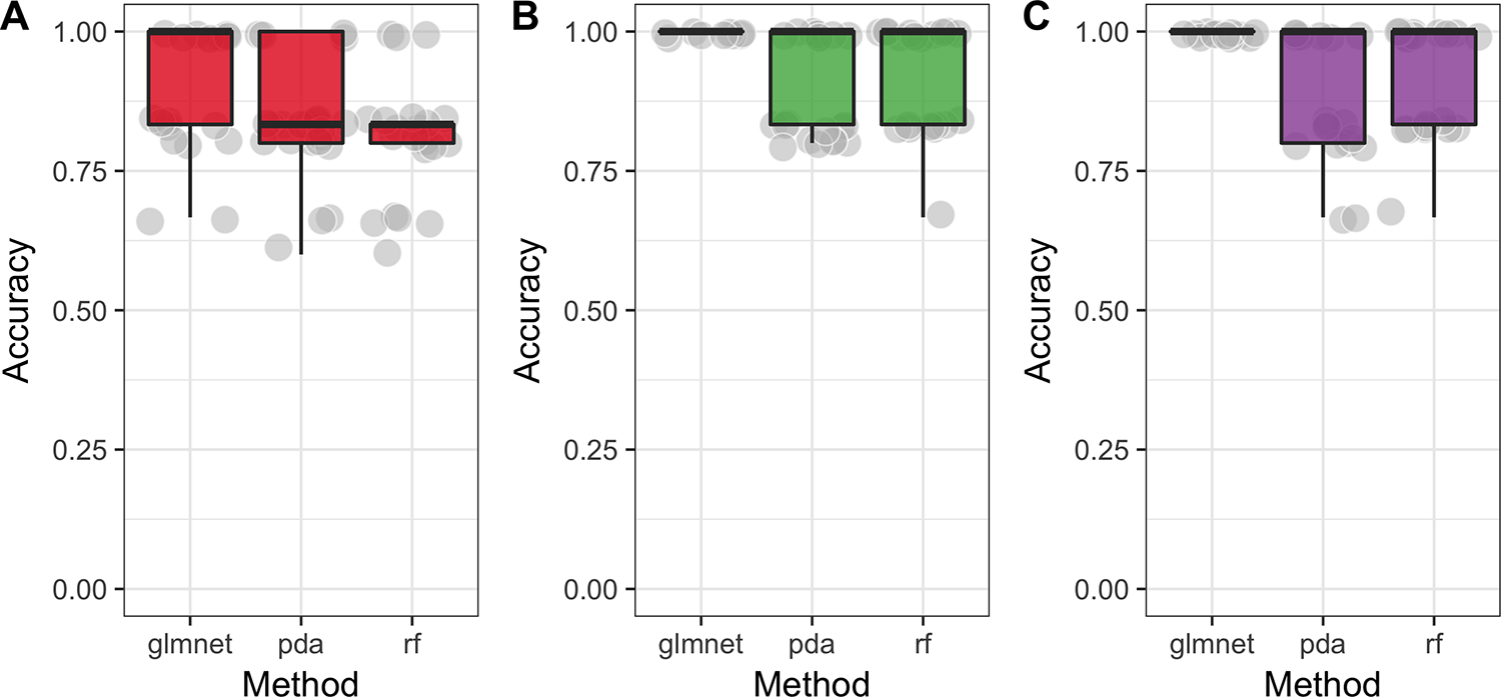
Averaged classification performance (accuracy) of three classifiers (rf = Random Forest, pda = Penalized Discriminant Analysis and glmnet = Lasso and Elastic-Net Regularized Generalized Linear Model) on data from untargeted analysis. Their performance was measured with cross-validation algorithm over 5 folds and 5 repetitions on data obtained with negative mode (A), positive mode (B) and a combination of two modes (C). We can see that on average all three classifiers show high accuracy (for all about 90% on average). Hence, it is possible to conclude that metabolites in untargeted analysis indeed have a discriminative power.

**Fig 7:**
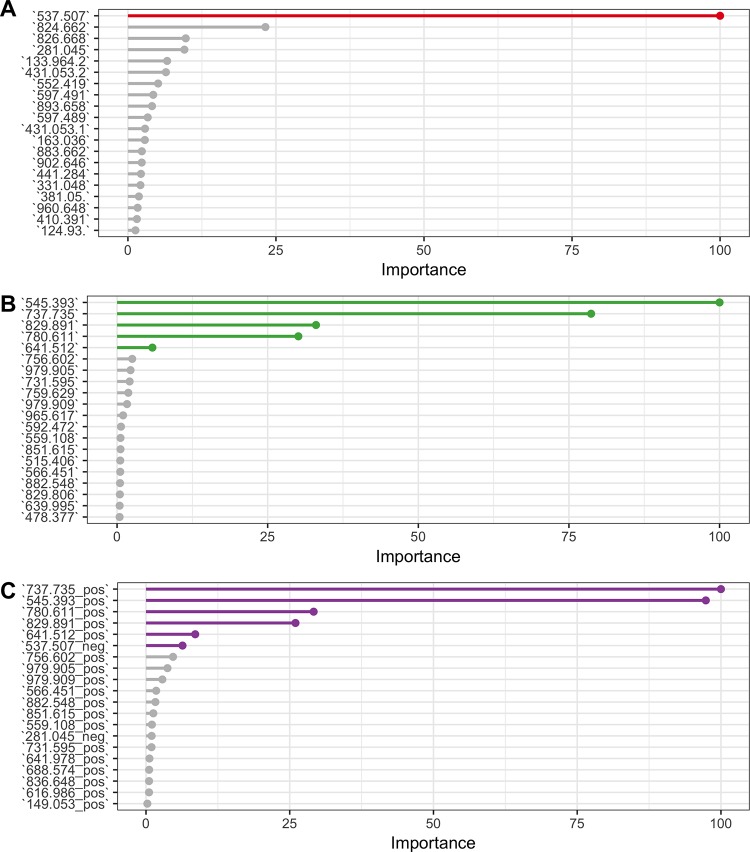
**Top 20 of the most influential metabolites used by the Random Forest algorithm trained on data obtained from negative mode (A), positive mode (B) and a combination of two modes (C).** Metabolites identified as significantly different from untargeted analysis (highlighted) are at the top of the list that was used by Random Forest classifier to obtain highly accurate classification performance.

For untargeted analysis all three machine-learning models show high accuracy (~90%) on all three modes (negative, positive and combined) (Fig 6). Metabolites that were identified using statistical tests in untargeted analysis were ranked first by the RandomForest algorithm in all three modes (Fig 7), suggesting that these metabolites carry true discriminative power between classes. Also, the constructed PCA plot based on significant metabolites clearly shows the separation between classes of samples (Fig 4).

## Discussion

Acylcarnitines are fatty acid and carnitine esters formed in the cytosol to transport fatty acids into the mitochondrial matrix for β-oxidation. The origins of plasma acylcarnitines, namely acetylcarnitine and propionylcarnitine, are mostly from the liver and can be transported throughout the body to wherever they are needed for energy production [25]. Acetylcarnitine is needed for the carnitine-dependent production of energy from different fatty acids and cell membrane structure maintenance [26]. Patients with atopic dermatitis had a significantly lower mean serum value for C2. Three carnitine ratios X.C2.C3….C0, C2…C0, Total.AC…C0 are all indicators of overall β-oxidation activity and the means are all lower in the blood serums of patients with atopic dermatitis. This means that the metabolites central in β-oxidation (acetyl-CoA and propionyl-CoA) have lower levels in AD patients’ serums. The decrease of C2 and C3 means that less ATP is produced through β-oxidation. The suppression of the β-oxidation and the accumulation of lipids in the liver due to atopic dermatitis has been demonstrated in mice by Seino *et al* [27]. To compensate the defective β-oxidation and the accumulation of lipids in the cytosol, ω- oxidation has been upregulated in AD patients, indicated by the higher level of Total.AC.DC…Total.AC discovered in this study. Dicarboxylacylcarnitines are the end products of ω- oxidation and in humans they are almost exclusively oxidized by peroxisomal fatty acyl-CoA oxidase of the classical β-oxidation pathway [28]. In addition, anaerobic glycolysis is also upregulated as compensation, indicated by the rise of lactate in AD patients [7]. The concentration of lactate in the serum is representative of the balance between the uptake and production of lactate in tissues whereas the normal levels range from 0.5 to 1.8mM. Lactate is formed by the reduction of pyruvate and metabolized by oxidation to pyruvate. This reaction is catalyzed by the NAD-dependent lactate dehydrogenase in the cytosol. Carbon dioxide and water are the end products of pyruvate metabolism and are part of the respiratory chain for energy production. Pyruvate is diverted to lactate when the production of pyruvate exceeds the capacity of oxidative metabolism [29]. Another indicator for energy compensation would be the decrease in blood glucose levels. Although the measured hexose levels in AD patients were lower in this study, the change was not statistically significant (p = 0.51).

Phosphocholines are the building blocks of cell membranes but they are also crucial to various lipid-signaling pathways. Ω-6 unsaturated fatty acids e.g. arachidonic acid form inflammatory prostanoids whereas anti-inflammatory and homeostatic prostanoids are derived from ω-3 unsaturated fatty acids. This can have an effect on systemic inflammation, allergy and asthma [30]. In this study we identified 3 different phosphatidylcholines that were downregulated in AD and one, PC(16:0–16:1) /(14:0–18:1) that was upregulated. Peiser *et al* demonstrated that patients with AD have a defective phosphatidylcholine-sphingomyelin transacylase [31] which might explain the higher concentration of that particular PC. Although it was not possible to determine whether the downregulated PCs from the targeted analysis were ω-3 or ω-6, the changes in measured levels clearly point to a disbalance in lipid signaling pathways that might have an effect on systemic inflammation. Treating AD patients with an IgE antibody omalizumab has been shown to alter the lipid profile [8] and bring them closer to normal levels. However the effects of various phosphatidylcholines and their roles in atopic dermatitis remain to be clarified.

The peptide discovered from the untargeted analysis in this study—DSGEGDFXAEGGGVR is a cleavage peptide of Fibrinogen A-α. The increase in the concentration of named peptide is not specific to atopic dermatitis but has also been seen in Buruli ulcer [32], tuberculosis [33] and diabetes [34]. The properties of fibrin clot in AD patients’ blood samples have been studied and the analysis revealed an increased clot mass and fiber thickness, faster clot formation among other altered plasma fibrin clot properties [35]. In another study, cutaneous fibrinolytic activity was noted in the acute phase of AD patients [36]. This might be one of the reasons why AD patients are at an increased risk for cardiovascular diseases (CVD)[37]. Other risk factors include the higher consumption of alcohol, increased smoking, obesity and less physical activity [38]. Increased short-chain dicarboxylcarnitine levels, also discovered in this study, have been found to correlate with CVD [39, 40]. The combination of lifestyle choices and serum biomarkers contribute to the higher risk for cardiovascular diseases in atopic dermatitis patients.

All statistically significant metabolites were confirmed to be relevant using machine-learning approaches. Although the repeated cross-validation technique was used which is designed to help avoid overfitting, the limited size of our dataset could be a source of unwanted bias that might potentially influence the results. To further confirm the findings in this study, a larger sample size of patients with atopic dermatitis and controls will be necessary in the future.

## Conclusion

Atopic dermatitis is a complex disease that has an impact beyond the lesions on the skin. The disbalance of serum metabolites including phosphatidylcholines, acylcarnitines and peptides all contribute to the changes seen in atopic dermatitis. This paper on the metabolomics of AD definitely contributes to the better understanding of the disease through the exploration of many disease-characteristic metabolites, both novel and old. These findings could potentially lead to a systemic explanation of atopic dermatitis cooperatively with genetics, proteomics and different machine-learning algorithms.

## Supporting information

S1 Fig Comparison of fragmentation spectra of m/z 537.507 to PC(16:0–16:1) /(14:0–18:1) in negative mode. (PNG)

S2 Fig Comparison of fragmentation spectra of 737.735 to DSGEGDFXAEGGGVR in positive mode. (PNG)

S3 Fig Comparison of fragmentation spectra of 780.611 to PC(16:1/20:4) in positive mode. (PNG)

S4 Fig Heatmap of significantly different metabolites from targeted analysis. First half of the columns belong to control samples, while the second part of the columns belong to patients. The first metabolite has a higher concentration in patients than in controls, while other metabolites on average have higher concentrations in controls than in patients.
(TIFF)

S5 Fig Heatmap of significantly different metabolites from untargeted analysis. The first half of the columns belong to control samples, while the second part of the columns belong to patients. The first two metabolites have a higher concentration in patients than in controls, while other metabolites on average have higher concentrations in controls than in patients.
(TIFF)

S6 Fig PLSDA for targeted analysis. Red circles–cases, blue squares–controls. A clear separation into clusters is not visible and an overlap of samples can be noted.
(TIFF)

S7 Fig **PLSDA for untargeted analysis based on metabolites identified as significant in (A) positive and (B) combined datasets.** Red circles–cases, blue squares–controls. Both plots show a clear separation of groups, which is confirmed by the performance of machine learning methods in Fig 5.
(TIFF)

S1 File Atopic dermatitis targeted.zip. Data table of results from targeted analysis.
(ZIP)

S2 File Atopic dermatitis untargeted.zip. Data table of results from untargeted analysis.
(ZIP)
