# Supplementary figures and images for: Blood serum metabolome of atopic dermatitis: Altered energy cycle and the markers of systemic inflammation
Source: PLoS One. 2017 Nov 27;12(11):e0188580. doi: 10.1371/journal.pone.0188580 (PMC5703555; doi:10.1371/journal.pone.0188580)

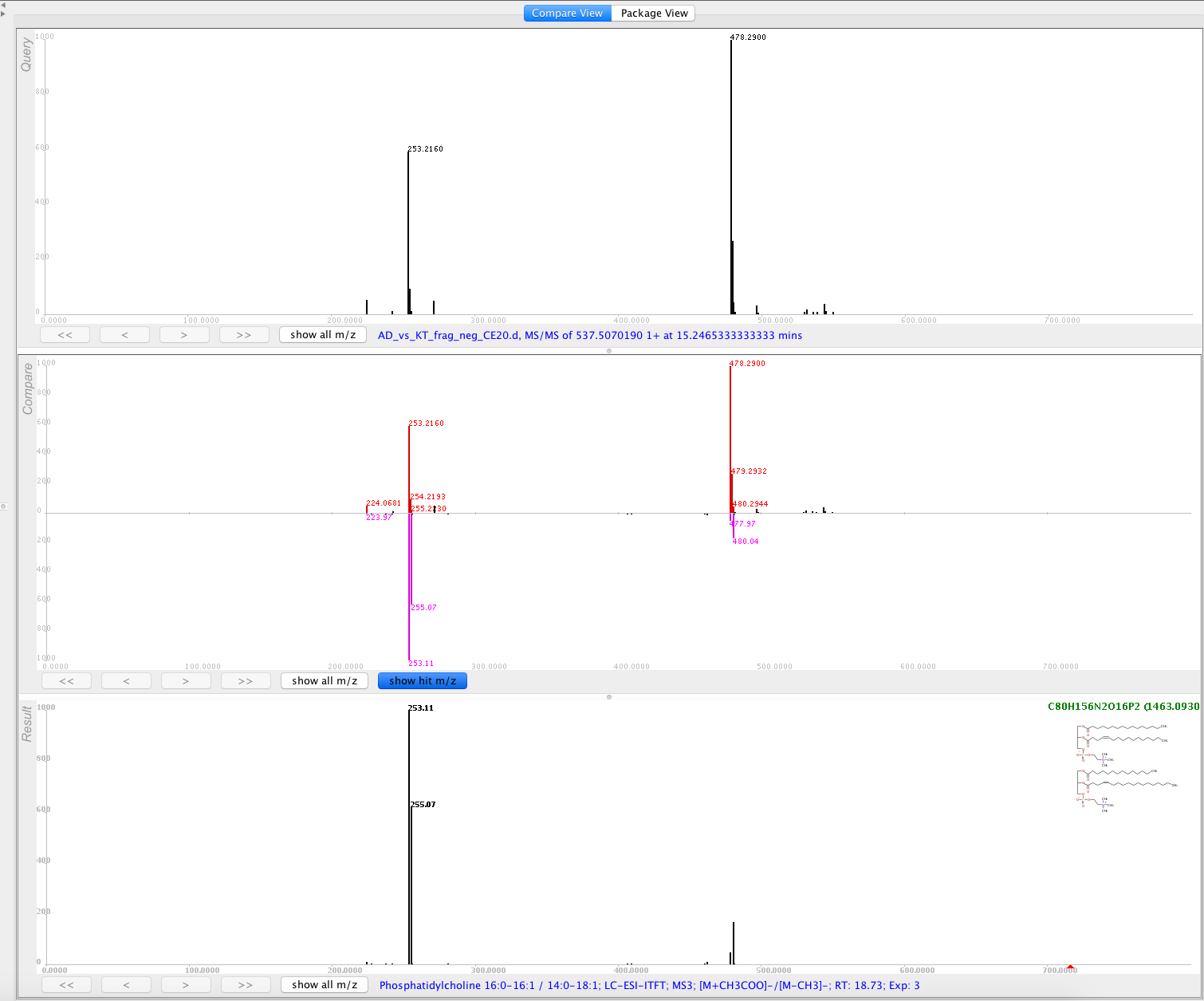

Supplement: S1 Fig — (PNG) [file pone.0188580.s001.png]

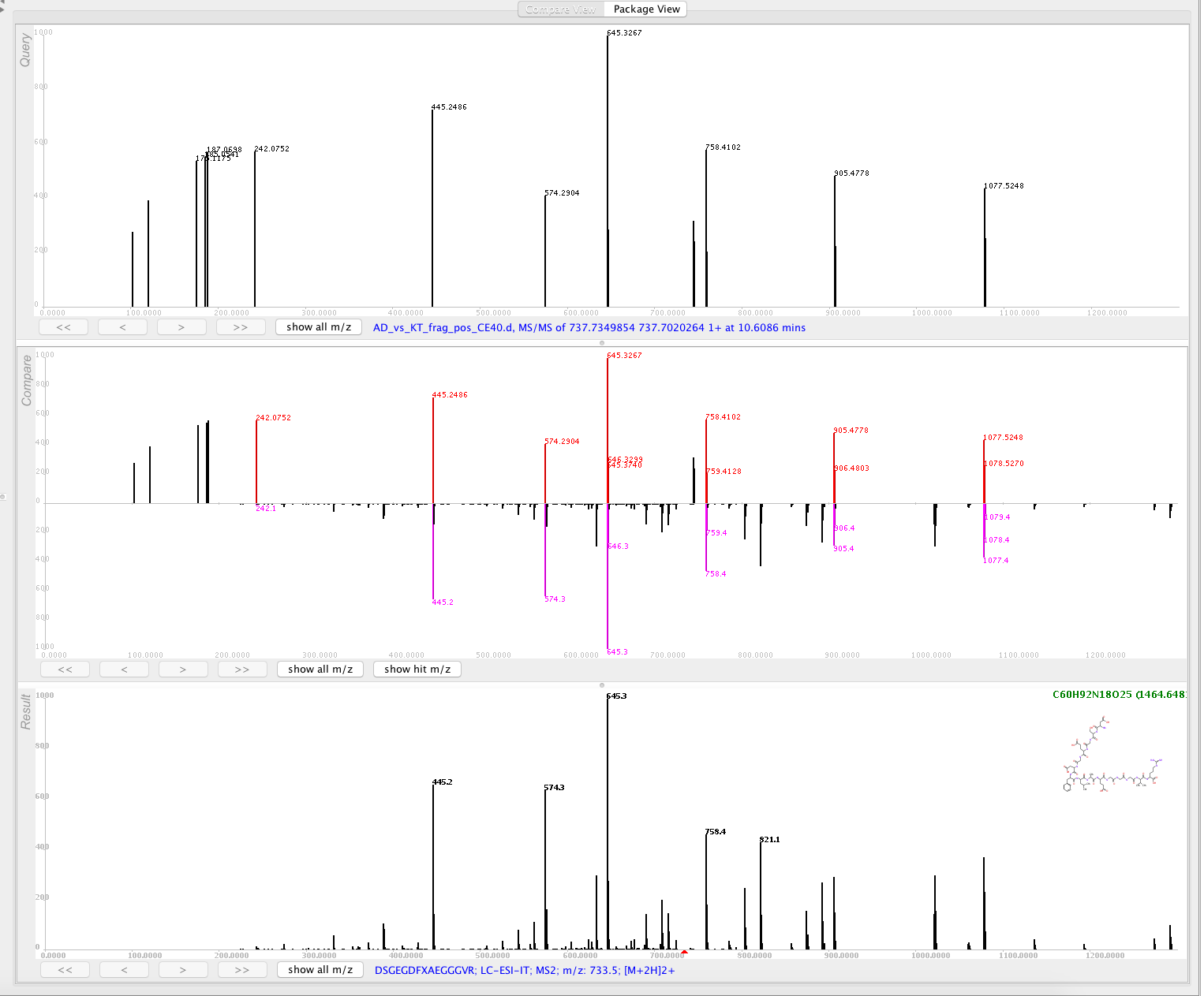

Supplement: S2 Fig — (PNG) [file pone.0188580.s002.png]

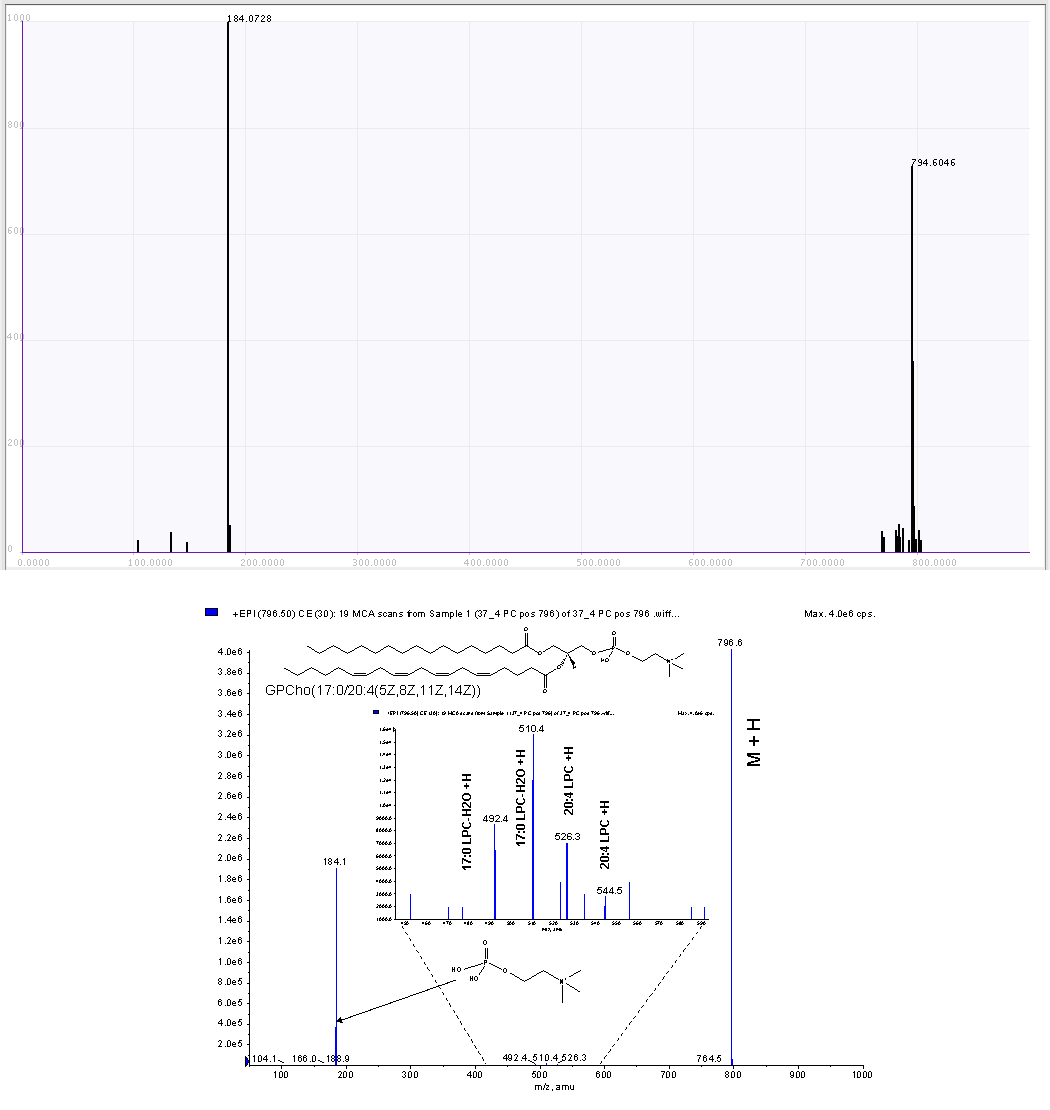

Supplement: S3 Fig — (PNG) [file pone.0188580.s003.png]

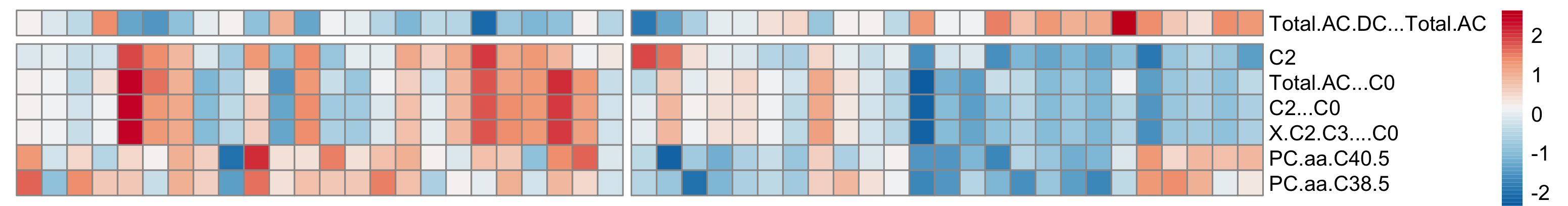

Supplement: S4 Fig — First half of the columns belong to control samples, while the second part of the columns belong to patients. The first metabolite has a higher concentration in patients than in controls, while other metabolites on average have higher concentrations in controls than in patients. (TIFF) [file pone.0188580.s004.tiff]

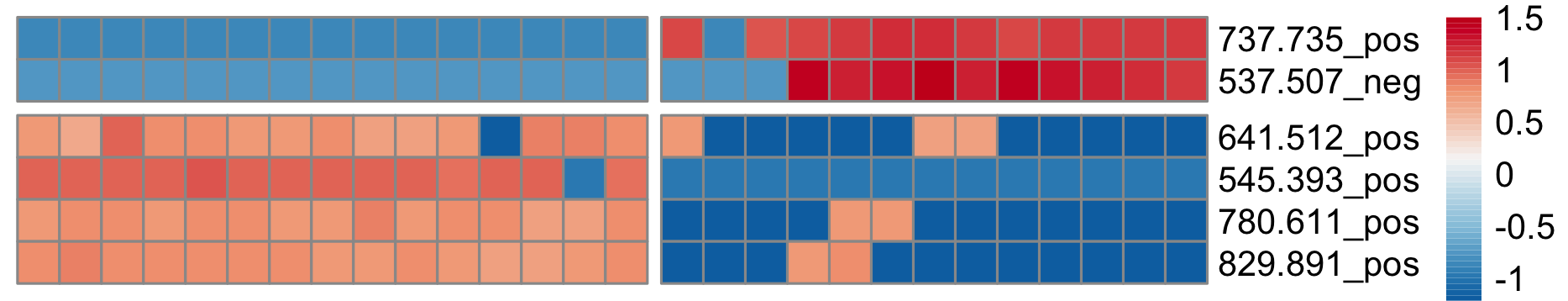

Supplement: S5 Fig — The first half of the columns belong to control samples, while the second part of the columns belong to patients. The first two metabolites have a higher concentration in patients than in controls, while other metabolites on average have higher concentrations in controls than in patients. (TIFF) [file pone.0188580.s005.tiff]

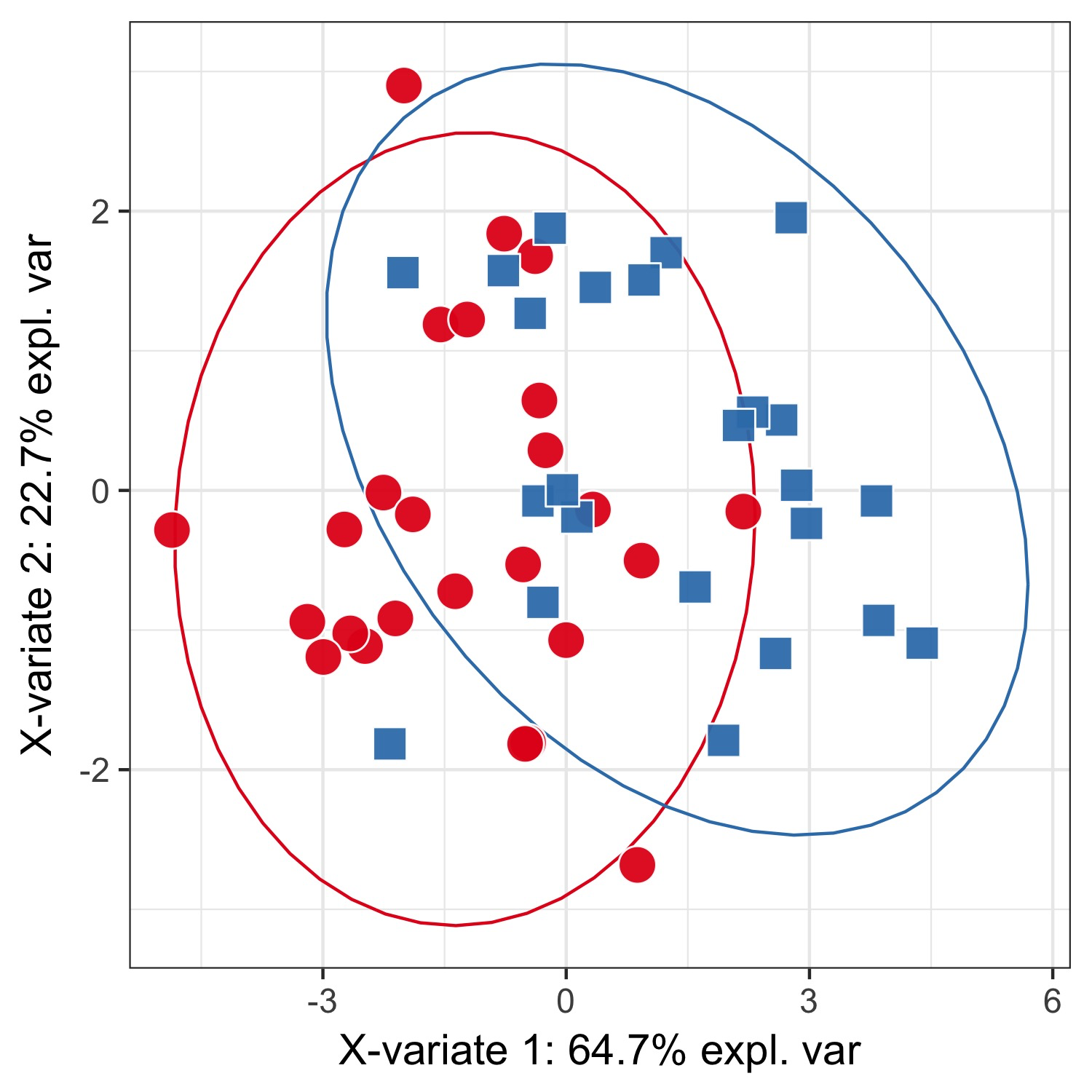

Supplement: S6 Fig — Red circles–cases, blue squares–controls. A clear separation into clusters is not visible and an overlap of samples can be noted. (TIFF) [file pone.0188580.s006.tiff]

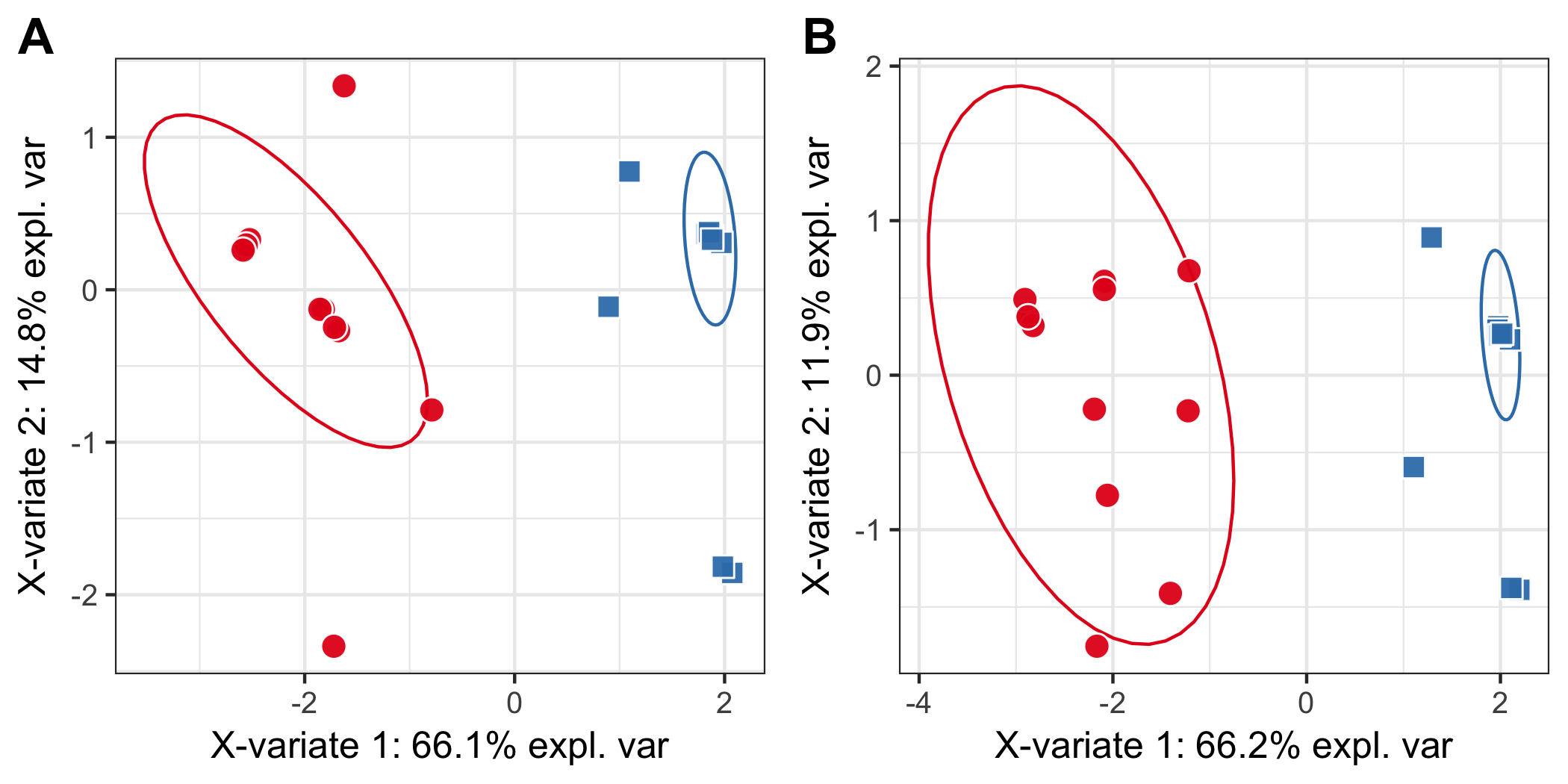

Supplement: S7 Fig — PLSDA for untargeted analysis based on metabolites identified as significant in (A) positive and (B) combined datasets. Red circles–cases, blue squares–controls. Both plots show a clear separation of groups, which is confirmed by the performance of machine learning methods in Fig 5. (TIFF) [file pone.0188580.s007.tiff]
